# PRMT5-TRIM21 interaction regulates the senescence of osteosarcoma cells by targeting the TXNIP/p21 axis

**DOI:** 10.18632/aging.102760

**Published:** 2020-02-05

**Authors:** Yu-Hang Li, Kui-Leung Tong, Jun-Lei Lu, Jie-Bin Lin, Zhen-Yan Li, Yuan Sang, Abdelmoumin Ghodbane, Xue-Juan Gao, Man-Seng Tam, Chang-Deng Hu, Huan-Tian Zhang, Zhen-Gang Zha

**Affiliations:** 1 Institute of Orthopedic Diseases and Department of Bone and Joint Surgery, The First Affiliated Hospital, Jinan University, Guangzhou 510630, Guangdong, China; 2 Key Laboratory of Functional Protein Research of Guangdong Higher Education Institutes, College of Life Science and Technology, Jinan University, Guangzhou 510632, Guangdong, China; 3 Department of Orthopedics, The Third Affiliated Hospital, Guangzhou University of Chinese Medicine, Guangzhou 510405, Guangdong, China; 4 Department of Orthopaedic Surgery, The Third Affiliated Hospital, Sun Yat-sen University, Guangzhou 510000, Guangdong, China; 5 IAN WO Medical Center, Macao Special Administrative Region, Macao 999078, China; 6 Department of Medicinal Chemistry and Molecular Pharmacology, Purdue University, West Lafayette, IN 47907, USA; 7 Purdue University Center for Cancer Research, Purdue University, West Lafayette, IN 47907, USA

**Keywords:** PRMT5, TRIM21, senescence, TXNIP, p21

## Abstract

Osteosarcoma (OS) is the most common bone malignancy in adolescents and has poor clinical outcomes. Protein arginine methyltransferase 5 (PRMT5) has recently been shown to be aberrantly expressed in various cancers, yet its role in OS remains elusive. Here, we found that PRMT5 was overexpressed in OS and its overexpression predicted poor clinical outcomes. PRMT5 knockdown significantly triggered pronounced senescence in OS cells, as evidenced by the increase in senescence-associated β-galactosidase (SA-β-gal)-stained cells, induction of p21 expression, and upregulation of senescence-associated secretory phenotype (SASP) gene expression. In addition, we found that PRMT5 plays a key role in regulating DNA damaging agents-induced OS cell senescence, possibly, via affecting the repair of DNA damage. Furthermore, we found that TXNIP acts as a key factor mediating PRMT5 depletion-induced DNA damage and cellular senescence. Mechanistically, TRIM21, which interacts with PRMT5, was essential for the regulation of TXNIP/p21 expression. In summary, we propose a model in which PRMT5, by interaction with TRIM21, plays a key role in regulating the TXNIP/p21 axis during senescence in OS cells. The present findings suggest that PRMT5 overexpression in OS cells might confer resistance to chemotherapy and that targeting the PRMT5/TRIM21/TXNIP signaling may enhance the therapeutic efficacy in OS.

## INTRODUCTION

Osteosarcoma (OS) is the most common primary bone malignancy and occurs primarily in children and adolescents [1, 2]. Chemotherapy such as cisplatin (CDDP) pre- and post- operative is applied as a standard treatment for those who are not suitable for surgical intervention. Despite significant advances in neoadjuvant chemotherapy, the prognosis of OS has barely improved in the past few decades [3]. Extensive studies have focused on genetic mutations of transcription factors, including p53 and Rb1, yet little is known about how druggable enzymes are involved in OS [2]. Recently, several epigenetic enzymes such as DNA methyltransferase 1 (DNMT1), enhancer of zeste homolog 2 (EZH2) and nuclear receptor-binding SET domain-containing (NSD), have been demonstrated to play a crucial role in OS, and targeting these enzymes enhanced OS cell apoptosis and chemosensitivity [4–6]. Therefore, targeting these enzymes in combination with chemotherapy might shed light on the treatment of OS.

Protein arginine methyltransferase 5 (PRMT5) is a druggable enzyme that has been shown to be aberrantly expressed in various cancers, and it acts as a putative oncogene in maintaining cancer cell survival [7–9], yet its role in OS remains elusive. We previously demonstrated that upstream signaling molecules, such as the protein kinase C (PKC), nuclear transcription factor Y (NF-Y), and carboxyl terminus of heat shock cognate 70-interacting protein (CHIP), play a key role in regulating PRMT5 expression and thus cell proliferation [10, 11]. Studies have also suggested that PRMT5 inhibition or loss-of-function impairs the DNA damage response (DDR) and induces apoptotic cell death via targeting p53, flap structure-specific endonuclease 1 (Fen1), and RuvB-like AAA ATPase 1 (RUVBL1), and Rad9 [12–16]. We have recently shown that PRMT5 functions as a master epigenetic activator of DDR genes, and that targeting PRMT5 by genetic knockdown or pharmacological inhibition can sensitize multiple cancer cell lines to radiation and chemotherapy [17]. PRMT5 is also responsible for regulating cellular senescence in glioblastoma neurospheres via Akt and ERK signaling [18].

Cellular senescence is a state of permanent cell cycle arrest characterized by an accumulation of senescence-associated β-galactosidase (SA-β-gal) and the appearance of a senescence-associated secretory phenotype (SASP). Several stimuli, such as oxidative stress and DNA damage, have been demonstrated to cause cellular senescence [19, 20]. The process of senescence is coordinated through two canonical pathways, the p53/p21 and Rb1/p16 pathways, yet cells can also undergo senescence via p53-independent pathways [21]. In addition, thioredoxin-interacting protein (TXNIP) has been demonstrated to functions as another key regulator of cellular senescence [22]. Although induction of senescence could be associated with drug resistance or tumor recurrence [23], there is no doubt that induction of cellular senescence is a promising antitumor mechanism either during cancer progression or within the chemotherapeutic windows in various cancers at the very early stage [24, 25].

In the present study, we found that PRMT5 is highly expressed in OS tissues and that its overexpression predicts poor clinical outcomes. Knockdown of PRMT5 induces pronounced senescence in OS cells while overexpression of PRMT5 in OS cells inhibits DNA damaging agents-induced senescence, presumably through a mechanism of regulating the DDR. Finally, we elucidated that TRIM21, an E3 ubiquitin-protein ligase, by interacting with PRMT5, plays a key role in regulating the TXNIP/p21 axis during senescence in OS cells.

## RESULTS

### PRMT5 is highly expressed in OS tissues, and its expression is correlated with OS clinicopathological features

**Figure 1:**
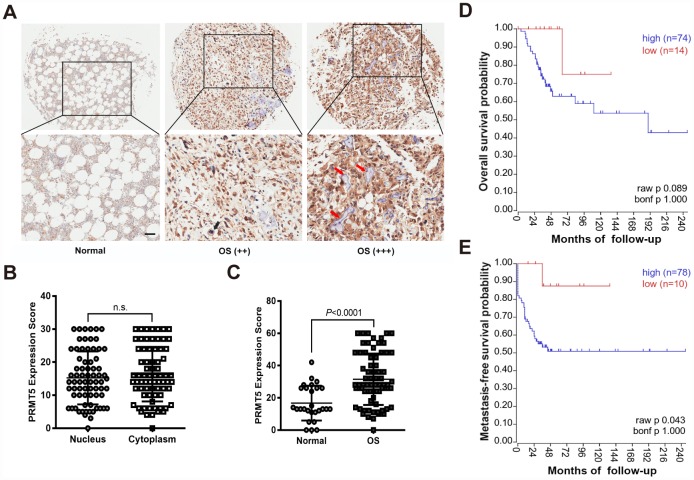
**PRMT5 is overexpressed in OS tissues and its expression predicts poor survival probability.** (**A**) The expression of PRMT5 was examined by IHC using commercial tissue microarrays (TMAs), which contained normal bone and different TNM stages of OS (++ indicated T1; +++ indicated T2). Representative images of PRMT5 expression in the tissues are shown. The red arrows indicate the trabecular bone. Scale bar = 50 μm. (**B**) The expression scores of PRMT5 in the cytoplasm and nucleus of OS cells (n = 72). (**C**) The expression scores of PRMT5 in normal bone (n = 27) and OS (n = 72). (**D** and **E**) PRMT5 expression along with OS survival probability was analyzed using the mixed osteosarcoma-Kuijjer dataset in the R2 Genomics Analysis and Visualization Platform (http://r2.amc.nl).

PRMT5 has previously been shown to be overexpressed in multiple human cancers, including prostate, lung, and colon cancers [7, 8], yet its role in OS is under investigation. Thus, we first examined the expression level of PRMT5 in the commercial tissue microarrays (TMAs) consisting of 27 normal bone and 72 OS tissues by immunohistochemistry (IHC). As shown in Figure 1A–1C, PRMT5 expression was significantly increased with disease progression from normal bone to grade I OS (++) and grade II OS (+++). These results confirm that the expression of PRMT5 in OS tissues is higher than that in normal bone. We further analyzed the association of PRMT5 expression with clinicopathological characteristics in 34 OS patients. As shown in Table 1, the high expression level of PRMT5 was positively correlated with local recurrence/lung metastasis and tumor grading, while negatively correlated with the survival status in OS patients (no correlation with age, sex, primary location, or histological type). In agreement with our findings, the publicly available mixed osteosarcoma-Kuijjer dataset also revealed that the high expression level of PRMT5 was correlated with poor metastasis-free survival probability, although the overall survival probability was not significantly different (Figure 1D and 1E). Since Ki67 is the most widely used marker for assessing the level of malignancy and prognosis; we then aimed to correlate the expression of PRMT5 with Ki67 in OS tissues [26]. In fact, PRMT5 expression positively correlated with Ki67 expression in another set of OS tissues (Supplementary Figure 1A and 1B). These results collectively suggest that abnormal expression of PRMT5 may play a role in OS.

**Table 1:** Association of PRMT5 expression with the clinicopathological characteristics of 34 OS patients.

**Variable**	***N***	**Low PRMT5**	**High PRMT5**	***P* value**
**Age (years)**				
<20	20	7	13	0.643
>20	14	6	8	
**Sex**				
Male	20	8	12	0.8
Female	14	5	9	
**Primary location**				
Proximal tibia	17	7	10	
Proximal humerus	9	3	6	0.979
Proximal femur	3	1	2	
Others	5	2	3	
**Histological type**				
Conventional OS	11	5	6	0.549
Others	23	8	15	
**Local recurrence/Lung metastasis**				
Yes	19	4	15	0.020*
No	15	9	6	
**Survival status**				
Yes	18	14	4	0.042*
No	16	7	9	
**Grading**				
I and II	30	24	6	0.019*
III	4	1	3	

Notes: ^a^Grouping of age was performed according to the median.

Abbreviations: OS, osteosarcoma

### Downregulation of PRMT5 elicits senescence in OS cells

**Figure 2:**
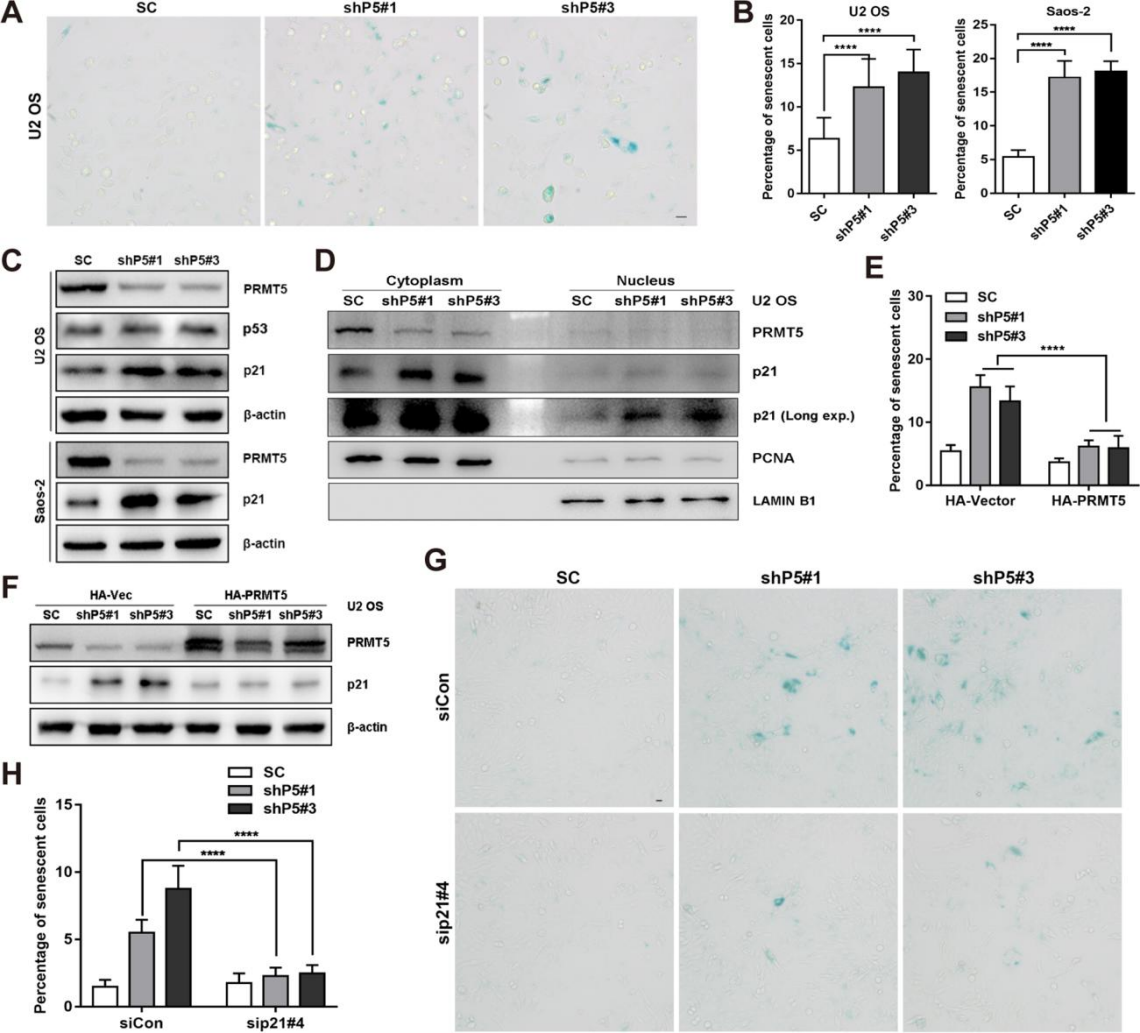
**Downregulation of PRMT5 elicits cellular senescence in OS.** (**A**) Two independent shRNAs targeting PRMT5 (shP5#1 and shP5#3) were applied to knock down PRMT5 expression in OS cell lines, and senescent cells were assessed using a SA-β-gal staining kit. Scale bar = 20 μm. (**B**) The percentage of senescent cells was quantified from three independent experiments, and the data are presented as the means ± SDs. ****, p< 0.0001. (**C**) The protein expressions of p53 and p21 with or without PRMT5 depletion in OS cells were determined by WB. (**D**) Cytoplasmic and nuclear proteins were prepared and then determined by WB. PCNA and LAMIN B1 were used as controls. (**E**) Plasmids encoding HA-PRMT5 were transfected into the SC, shP5#1 or shP5#3 U2 OS cells, and the percentage of senescent cells was quantified. ****, p< 0.0001. (**F**) Plasmids encoding HA-PRMT5 were transfected into SC, shP5#1 or shP5#3 U2 OS cells, the expressions of PRMT5 and p21 were then determined by WB. (**G**–**H**) siRNA targeting p21 (sip21#4) was transfected into SC, shP5#1 or shP5#3 U2 OS cells for 3 days, the senescent cells were visualized using a SA-β-gal staining kit. Scale bar = 10 μm. The percentage of senescent cells was quantified. ****, p< 0.0001.

Next, we sought to investigate the possible effects of PRMT5 on the growth of OS cells. As shown in Supplementary Figure 2A–2C, knockdown or inhibition of PRMT5 showed little effect on the apoptosis of U2 OS cells. However, knockdown of PRMT5 significantly increased the percentage of senescent cells and retarded the cell proliferation of OS, as evidenced by SA-β-gal staining, 5-Ethynyl-2'-deoxyuridine (EdU) incorporation assay, as well as the protein expression of p-mTOR and p-p70 S6K, which distinguish quiescence and senescence [27] (Figure 2A and 2B, Supplementary Figure 2D–2F). Senescent cells have been demonstrated to actively secrete a group of proteins named SASP [28]; and we confirmed that knockdown of PRMT5 upregulated the mRNA expression of SASP genes, including CXCL-1, CXCL-2, CXCL-3, IL-6, IL-8, TNF-α, ICAM-1, and CCL2 (Supplementary Figure 2G). Cellular senescence can be triggered by multiple pathways, including the p53-p21 and Rb-p16 axes [21, 28]. Since PRMT5 was previously demonstrated to play a key role in epigenetically silencing the transcription of p21 [29, 30], we then explore this in OS cells. Surprisingly, no significant change of p21/CDKN1A mRNA level was found upon PRMT5 depletion in the U2 OS cells (Supplementary Figure 2H). However, knockdown of PRMT5 dramatically increased the protein expression of p21 (but not p53) in the U2 OS cells (Figure 2C). Similar induction of p21 at the protein level was found in shP5#1 and shP5#3 Saos-2 cells, in which p53 expression is lost (Figure 2C). In addition, a marked increase of p21 expression at both the cytoplasm and nucleus was validated using subcellular fractionation and immunofluorescence analyses (Figure 2D, Supplementary Figure 2I).

In contrast, overexpression of PRMT5 by transiently transfection of the plasmid encoding HA-PRMT5 markedly reduced the percentage of senescent cells and the expression of p21 triggered by PRMT5 depletion, indicating the specific role of PRMT5 in regulating cellular senescence (Figure 2E and 2F, Supplementary Figure 2J). To explore whether p21 expression is essential for PRMT5 knockdown-triggered cellular senescence, we combined the application of the siRNA to interfere with the p21 expression (Supplementary Figure 2K). The knockdown of p21 significantly reduced the percentage of SA-β-gal stained-senescent cells in shP5 cells (Figure 2G and 2H). Altogether, these results consistently support a crucial role of PRMT5 in regulating p21 expression and thus the cellular senescence in OS.

### PRMT5 inhibits DNA damaging agents-induced OS cell senescence

**Figure 3:**
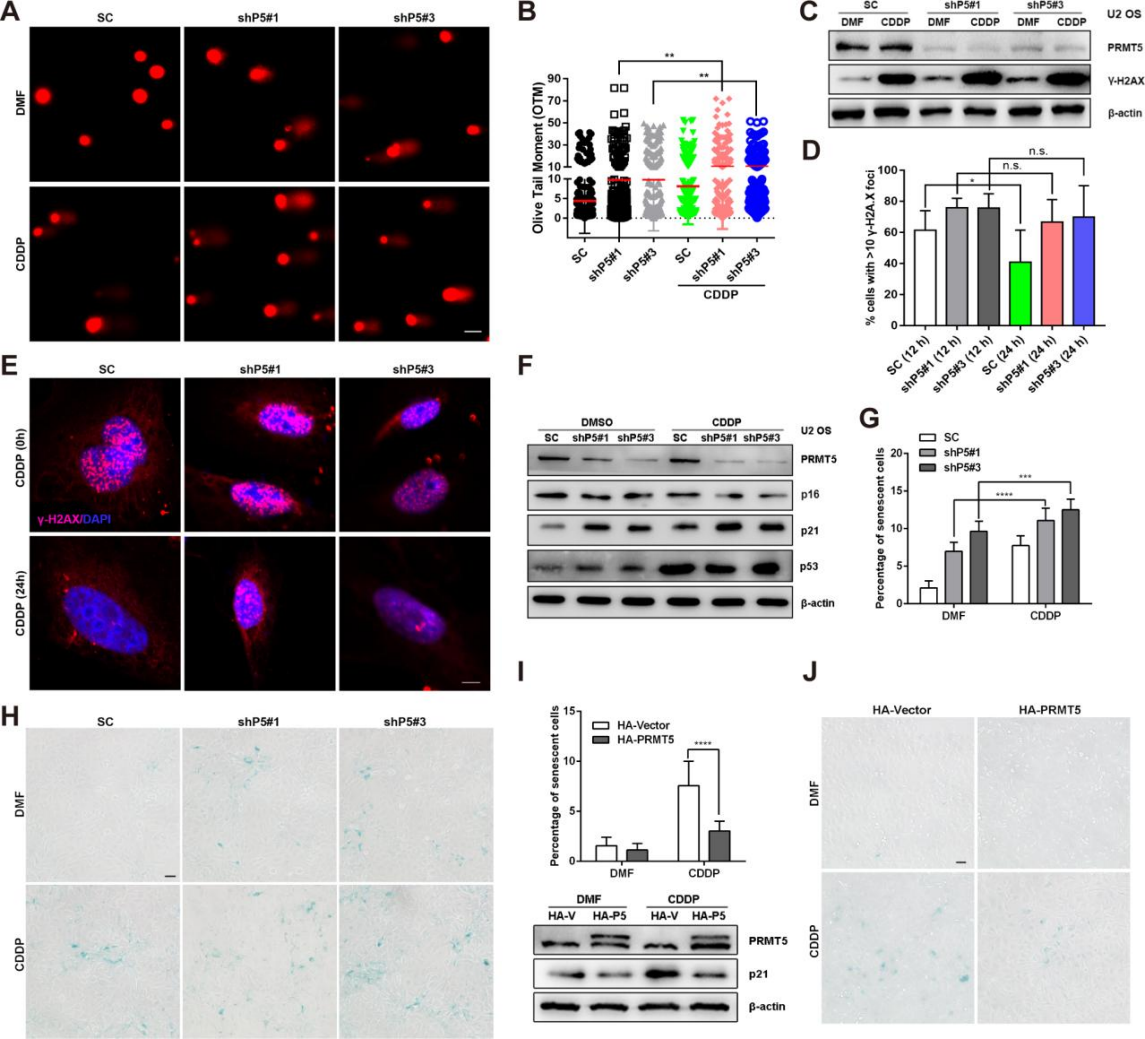
**PRMT5 inhibits DNA damaging agents-induced OS cell senescence.** (**A** and **B**) Cisplatin (CDDP, 10 μM) was added to SC, shP5#1, and shP5#3 U2 OS cells for 24 h. Then, DSBs were visualized by a comet assay, followed by quantification of the OTM with Open Comet software. Scale bar = 20 μm. **, *p*< 0.01. (**C**) SC, shP5#1 and shP5#3 U2 OS cells were treated with 10 μM CDDP for 24 h, the expressions of PRMT5 and γ-H2A.X were measured by WB. (**D**, **E**) SC, shP5#1 and shP5#3 U2 OS cells were treated with 20 μM CDDP for 3 h; the medium was then replaced with fresh medium, and cells were cultured for 12 h or 24 h (time for DNA repair). Antibody against γ-H2A.X was used for immunofluorescence staining, DAPI was used to counterstain the nucleus, and the percentage of positive cells (with ≥10 foci per nucleus considered positive) was counted in three independent experiments and quantified with ImageJ software. Scale bar = 10 μm. *, *p*< 0.05. (**F**) SC, shP5#1 and shP5#3 U2 OS cells were treated with 10 μM CDDP for 24 h, the expressions of PRMT5, p16, p21 and p53 were measured by WB. (**G**, **H**) SC, shP5#1 and shP5#3 U2 OS cells were treated with 10 μM CDDP for 12 h, the percentage of senescent cells was quantified. *, *p*< 0.05; ***, *p*< 0.001; the cellular senescence was visualized using a SA-β-gal staining kit. Scale bar = 50 μm. (**I**, **J**) U2 OS cells were transfected with plasmids encoding HA-PRMT5, followed by treated with CDDP for 12 h, and the percentage of senescent cells was quantified. ****, *p*< 0.0001; the expressions of PRMT5, TXNIP and p21 were determined by WB; the cellular senescence was visualized using a SA-β-gal staining kit. Scale bar = 50 μm.

Increasing evidence suggests that DNA damage is a common mediator of cellular senescence [31, 32]; therefore, we investigated whether the cellular senescence triggered by PRMT5 depletion in OS was associated with DNA damage. Knockdown of PRMT5 (shP5#1 or shP5#3) initiated obvious DNA double-strand breaks (DSBs) in U2 OS cells, as evidenced by the comet assay, which was used to visualize DNA fragmentation in individual cells. In addition, the Olive tail moment (OTM) indicated the tail length was increased in cells upon PRMT5 depletion (Figure 3A and 3B). This effect became more pronounced with the addition of CDDP, a first-line DNA damaging chemotherapeutic reagent for OS treatment [33] (Figure 3A and 3B). In support of this finding, the expression and percentage foci of γ-H2A.X (≥ 10), a marker of DNA DSBs, as reported previously [34], were increased after the PRMT5 knockdown (Figure 3C–3E). Since PRMT5 is reported to cause DNA damage and regulate DNA repair signaling [14–17], we sought to determine whether knockdown of PRMT5 affects DNA repair signaling in OS cells. As shown in Figure 3D–3E, the percentage of γ-H2A.X foci (≥ 10) positive cells in the scramble control (SC) group was noticeably decreased after 12 and 24 h of recovery from CDDP treatment (replacement with fresh medium), while the percentage of γ-H2A.X foci remained constant in the shP5#1 and shP5#3 groups, indicating the impairment of DNA repair signaling.

CDDP has been reported to induce cancer cells undergoing senescence [35, 36]. Next, we sought to investigate whether PRMT5 plays a role in CDDP-induced cellular senescence. Of note, induction of p21 accompanied with cellular senescence was elicited upon treatment of CDDP, while knockdown of PRMT5 further enhanced the p21 protein expression as well as OS cell senescence (Figure 3F–3H). On the contrary, overexpression of PRMT5 remarkably suppressed CDDP-induced p21 protein level and OS cell senescence (Figure 3I–3J). These results collectively suggest that PRMT5 overexpression in OS cells might confer resistance to CDDP via modulation of cellular senescence.

### TXNIP plays a role in regulating cellular senescence induced by PRMT5 knockdown

**Figure 4:**
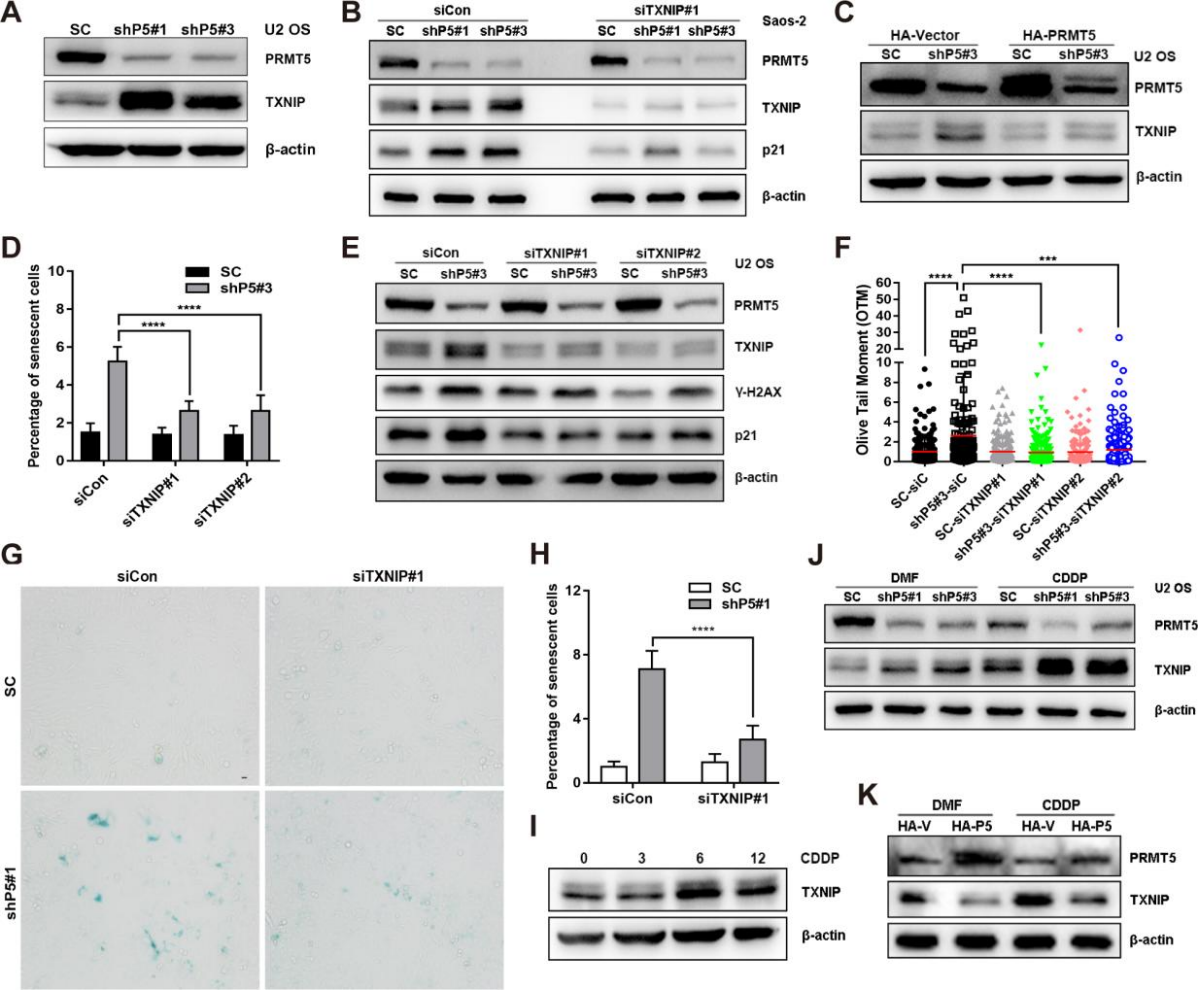
**TXNIP is essential for the induction of cellular senescence by PRMT5 depletion.** (**A**) The protein expression of TXNIP was determined by WB with or without PRMT5 knockdown in U2 OS cells. (**B**) siRNA targeting TXNIP was transfected into SC, shP5#1 or shP5#3 Saos-2 cells, and the expressions of PRMT5, TXNIP, and p21 were measured by WB; β-actin was used as the internal control. (**C**) Plasmids encoding HA-PRMT5 were transfected into SC or shP5#3 U2 OS cells, and the expression of PRMT5 and TXNIP was determined by WB. (**D**) Two independent siRNAs targeting TXNIP (siTXNIP#1 and siTXNIP#2) were transfected into SC or shP5#3 U2 OS cells for 3 days, the percentage of senescent cells was quantified. ****, *p*< 0.0001. (**E**) siRNAs targeting TXNIP were transfected into SC, shP5#1 or shP5#3 U2 OS cells, and the expressions of PRMT5, TXNIP, γ-H2A.X, and p21 were measured by WB; β-actin was used as the internal control. (**F**) DSBs were quantified by Open Comet software. ***, *p*< 0.001; ****, *p*< 0.0001. (**G**, **H**) siRNA targeting TXNIP was transfected into SC and shP5#1 U2 OS cells, and cellular senescence was visualized using a SA-β-gal staining kit. Scale bar = 10 μm. the percentage of senescent cells was quantified. ****, *p*< 0.0001. (**I**) U2 OS cells were treated with CDDP for different durations, the expression of TXNIP was measured by WB. (**J**) 10 μM CDDP was added to SC, shP5#1, and shP5#3 cells for 12 h, the expressions of PRMT5 and TXNIP were determined by WB. (**K**) U2 OS cells were transfected with plasmids encoding HA-PRMT5, followed by treated with CDDP for 12 h, the expressions of PRMT5 and TXNIP were determined by WB.

Next, we sought to identify the key factor mediating PRMT5 depletion-induced DNA damage and cellular senescence. TXNIP has recently been reported as a key regulator of oxidative stress, DNA damage, and cellular senescence [22]; thus, we explored the possible involvement of TXNIP in PRMT5 depletion-induced cellular senescence. Of significance, knockdown of PRMT5 upregulated the protein but not the mRNA expression level of TXNIP in the U2 OS cells (Figure 4A, Supplementary Figure 3A). A similar result was obtained using another OS cells, Saos-2 (Figure 4B). In contrast, overexpression of PRMT5 by transfecting the plasmid encoding HA-PRMT5 markedly abolished the induction of TXNIP by shP5#3 (Figure 4C). Since cytoplasmic TXNIP has been demonstrated to be highly correlated with cellular senescence [37], we also explored its localization and found that PRMT5 depletion led to a marked increase of both cytoplasmic and nuclear TXNIP (Supplementary Figure 3B and 3C). Functionally, knockdown of TXNIP by two different small interfering RNAs (siRNAs, siTXNIP#1 and siTXNIP#2) significantly reduced the percentage of senescent cells resulting from PRMT5 depletion (Figure 4D, Supplementary Figure 3D). In agreement with this finding, siTXNIP#1 and siTXNIP#2 also greatly suppressed the induction of p21 as well as the DSBs caused by shP5#3 (Figure 4E and 4F, Supplementary Figure 3E). Similarly, interfering TXNIP by siTXNIP#1 also remarkably reduced the percentage of SA-β-gal stained-senescent cells and the induction of p21 elicited by another shRNA of PRMT5, shP5#1 (Figure 4B, Figure 4G and 4H, Supplementary Figure 3F). Notably, we found that the treatment of OS cells with CDDP induced TXNIP expression at a time of 6 and 12 h, and this induction of TXNIP was enhanced upon PRMT5 depletion by shP5#1 and shP5#3, while suppressed by PRMT5 overexpression (Figure 4J, 4K). These results together suggest that TXNIP acts as a key factor mediating PRMT5 depletion-induced DNA damage and cellular senescence.

### TRIM21 interaction with PRMT5 is essential for the regulation of the TXNIP/p21 axis

**Figure 5:**
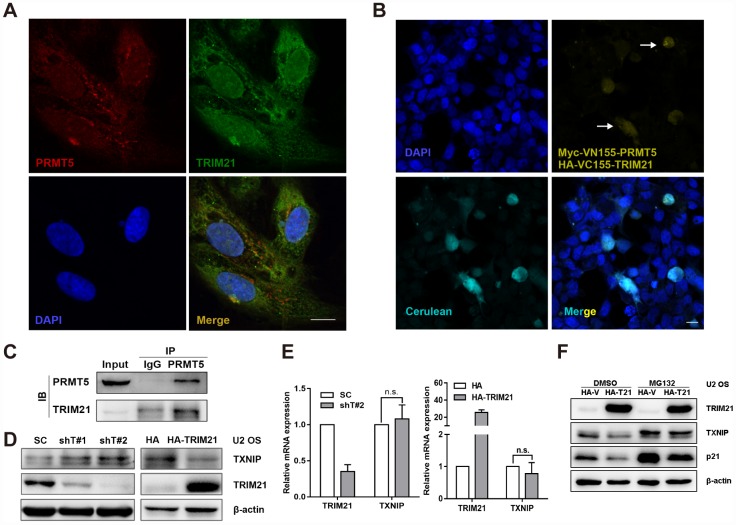
**TRIM21 interacts with PRMT5 in U2 OS cells.** (**A**) Colocalization of TRIM21 and PRMT5 was observed in U2 OS cells using antibodies against TRIM21 (green) and PRMT5 (red). Scale bar = 20 μm. (**B**) DAPI was used to indicate nuclei. Myc-VN155-PRMT5 and HA-VC155-TRIM21, along with HA-cerulean, were cotransfected into U2 OS cells for 48 h, and the reconstituted Venus fluorophore (yellow, arrows) was visualized via confocal microscopy. Scale bar = 20 μm. (**C**) The endogenous interaction between TRIM21 and PRMT5 was validated using a co-IP assay. (**D**, **E**) shRNAs targeting TRIM21 (shT#1 and shT#2) or plasmid encoding HA-TRIM21 were applied to knock down or overexpress TRIM21, and the protein and mRNA levels of TRIM21 and TXNIP were then determined by WB or quantitative real-time PCR, respectively. (**F**) Dox-inducible TRIM21-expressing cells was treated with MG132 (10 μM) for 12h, the protein expression of TRIM21, TXNIP, and p21 was then determined by WB.

It is well characterized that PRMT5 plays a key role in regulating transcription via methylating histone or non-histone substrates [7]. Given that PRMT5 has a marginal effect on the transcription of TXNIP and p21, we then hypothesized that PRMT5 might regulate TXNIP protein expression in an indirect manner, e.g., via modulating protein-protein interaction. We have previously identified TRIM21 as a potential interacting partner of PRMT5 by mass spectrometry in prostate cancer cells [11]. Next, we sought to assess whether TRIM21 is necessary for PRMT5-regulated TXNIP and p21 expression. Of significance, PRMT5 and TRIM21 were colocalized in the cytoplasm in U2 OS cells (Figure 5A), and bimolecular fluorescence complementation (BiFC) assay, an imaging method for visualization of protein-protein interactions in living cells [38, 39], confirmed their interaction in the U2 OS cells (Figure 5B). The interaction between PRMT5 and TRIM21 was further validated by the coimmunoprecipitation (co-IP) assay (Figure 5C). Thus, these results demonstrate that PRMT5 interacts with TRIM21 in the U2 OS cells. Next, shTRIM21 stable cells or doxycycline (Dox)-inducible H125-TRIM21 expressing cells were established for further studies. As shown in Figure 5D and 5E, knockdown of TRIM21 negatively regulated the expression of TXNIP at the protein level but not at the mRNA level. In contrast, overexpression of TRIM21 significantly attenuated TXNIP protein level without an obvious effect on TXNIP transcription (Figure 5D and 5E). In addition, the treatment of MG132 (proteasome inhibitor) partially restored TXNIP (p21 as positive control) expression even in the presence of HA-TRIM21 (Figure 5F). These results suggest that TRIM21 can post-translationally regulate the expression of TXNIP.

**Figure 6:**
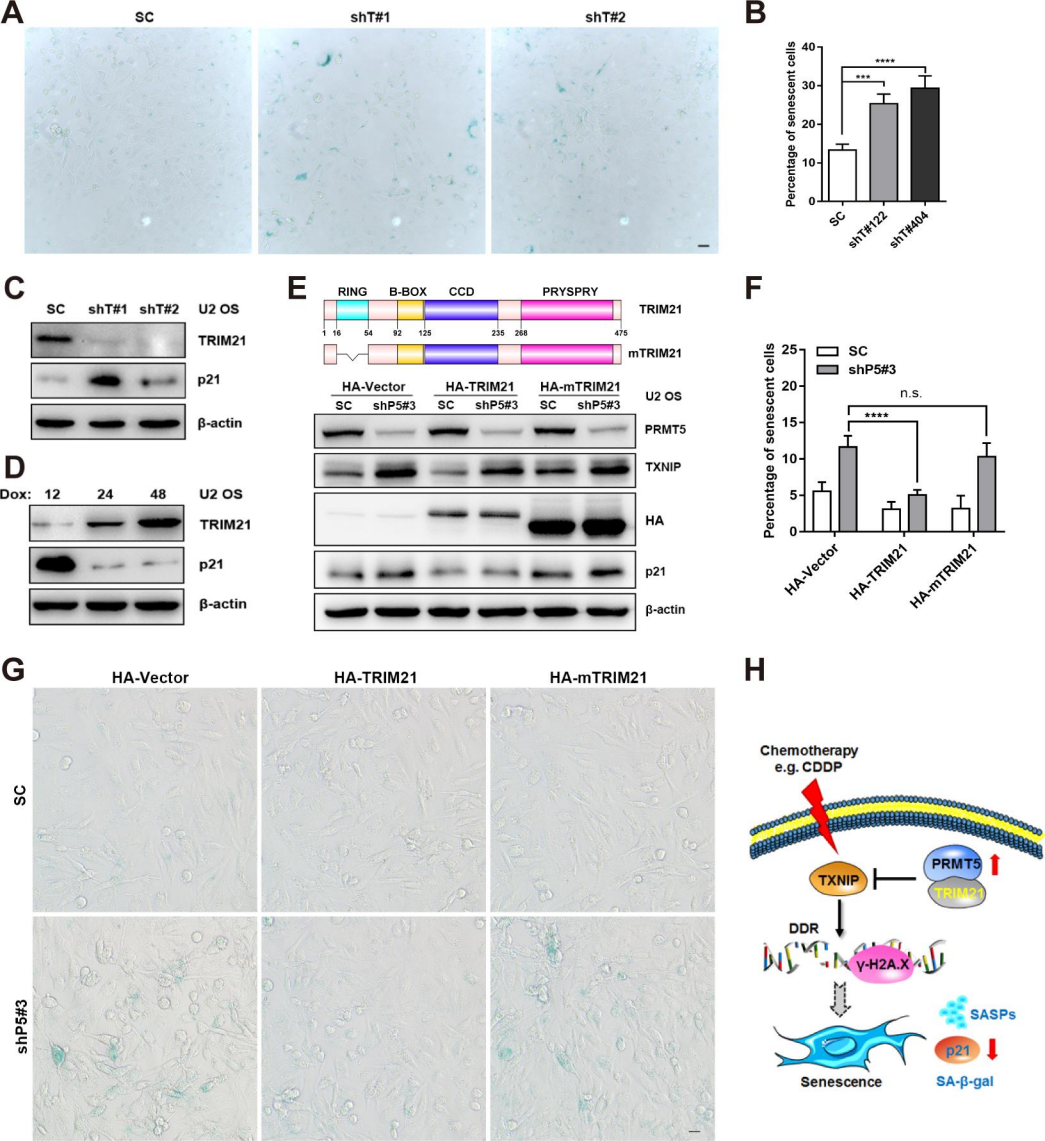
**TRIM21 is required for the regulation of the TXNIP/p21 axis by PRMT5.** (**A**) Two independent shRNAs targeting TRIM21 (shT#1 and shT#2) were utilized to knock down TRIM21, and cellular senescence was visualized using a SA-β-gal staining kit. Scale bar = 20 μm. (**B**) The percentage of senescent cells was quantified. ***, *p*< 0.001; ****, *p*< 0.0001. (**C**) The protein expression of p21 with or without TRIM21 depletion was determined by WB. (**D**) U2 OS cells expressing Dox-inducible HA-TRIM21 were established; the cells were induced with Dox for different durations, and p21 expression was then measured by WB. (**E**) Plasmids expressing HA-TRIM21 or the HA-TRIM21 ∆RING mutant (HA-mTRIM21) were transfected into SC or shP5#3 U2 OS cells, and the protein expression of PRMT5, TXNIP, and p21 was then determined by WB. (**F**–**G**) Plasmids expressing HA-TRIM21 or the HA-TRIM21 ∆RING mutant (HA-mTRIM21) were transfected into SC or shP5#3 cells, and cellular senescence was visualized using a SA-β-gal staining kit. Scale bar = 20 μm; the percentage of senescent cells was quantified, and the data are presented as the means ± SDs. n.s., no significance; ****, *p*< 0.0001. (**H**) Schematic depicting the involvement of the PRMT5/TRIM21 complex in regulation of the DDR and cellular senescence via the TXNIP/p21 axis in U2 OS cells.

Next, we aimed to explore the possible role of TRIM21 in PRMT5 depletion-regulated TXNIP/p21 axis in the U2 OS cells. As shown in Figure 6A–6C, knockdown of TRIM21 increased the percentage of senescent cells, accompanied by a significant induction of p21 expression. Conversely, enforced expression of TRIM21 by Dox induction markedly decreased p21 expression (Figure 6D). Further, we investigated the functional domain of TRIM21 in regulating TXNIP/p21 expression and the OS cell senescence. As shown in Figure 6E, overexpression of HA-TRIM21 remarkably attenuated the induction of TXNIP and p21 in the shP5#3 cells. Nevertheless, a comparable expression of TXNIP and p21 in the SC and shP5#3 cells was observed either by transfecting the cells with the HA-Vector or HA-mTRIM21, a RING domain deleted mutant which lacks E3 ubiquitin ligase activity (Figure 6E). Consistently, overexpression of HA-TRIM21 but not HA-mTRIM21 remarkably abolished PRMT5 knockdown-induced cellular senescence (Figure 6F and 6G). Taken together, our results suggest that TRIM21 functional interacting with PRMT5 is involved in the regulation of the TXNIP/p21 axis, and the DNA damaging agent-induced cellular senescence (Figure 6H).

## DISCUSSION

In recent years, PRMT5, as an oncoprotein, has gained increasing attention in terms of cancer prevention and therapy. PRMT5 is aberrantly expressed in various cancers and that inhibition or knockdown of PRMT5 suppresses cancer cell proliferation, induces cell cycle arrest, and abolishes cancer metastasis both *in vitro* and *in vivo* [7, 8]. In the present study, we first found that PRMT5 expression was positively correlated with OS pathological grade, establishing the clinical importance of PRMT5 expression in OS.

Recent studies have suggested that induction of cellular senescence is a promising strategy for enhancing therapeutic efficacy in various cancers, including OS [23, 25, 40]. We then explored whether PRMT5 plays a role in regulating senescence of OS cells with or without the DNA damaging stress elicited by the most common first-line chemotherapeutic drug, CDDP. Several lines of evidence supporting the fact that PRMT5 is essential for regulating CDDP-induced OS cell senescence: firstly, at the basal level, PRMT5 knockdown significantly increases the number of senescent cells in OS and induces the SASPs gene expression, through a mechanism involving p21 expression; while ectopic expression of PRMT5 suppresses PRMT5 knockdown-induced cellular senescence. Our finding is in according to a recent study elucidating that PRMT5 mediates the activation of Akt and ERK in glioblastoma neurosphere cells by regulating PTEN expression and ultimately participates in cellular senescence [18]. Secondary, although CDDP has been demonstrated to induce cancer cells into senescence through sequential activation of the DNA damage response and the p53/p21 pathway in the literature [20, 35], and in our hands, knockdown of PRMT5 was found to further promote CDDP-induced p21 expression and senescent OS cells, while PRMT5 overexpression remarkably diminished CDDP-induced p21 protein level and senescent cells. Thirdly, since DNA damage is the most important inducer of cellular senescence [41–43], we also reveal the possible mechanism demonstrating that PRMT5 plays a crucial role in regulating DSBs and DDR at either basal level or in CDDP-treated levels. This result is consistent with our recent publication reporting that PRMT5 functions as a master epigenetic activator of DDR genes [17]. These results together suggest that overexpression of PRMT5 may confer resistance of OS cells to CDDP by regulating cellular senescence, and that targeting the druggable PRMT5 in combination with chemotherapy may be utilized as a strategy for OS treatment.

PRMT5 performs its function mainly through epigenetic silencing or direct methylation of histone and nonhistone molecules [7]. A series of studies have reported that PRMT5 regulates p21 expression via an epigenetic silencing mechanism [29, 30], yet in our study, knockdown of PRMT5 did not significantly alter the transcription of p21 and that the induction of p21 protein expression by PRMT5 depletion is not dependent on p53. Interestingly, we identified TXNIP as a critical regulator of senescence in OS cells. TXNIP is expressed at a lower level in various cancers (such as liver, breast and bladder cancer) and is a multifunctional protein that controls various cellular processes, such as cell proliferation, apoptotic signaling, oxidative stress and inflammation [44–47]. Consistent with the findings that overexpression of TXNIP promotes DNA damage in esophageal adenocarcinoma [48], senescence in vascular endothelial cells [49], we demonstrated that TXNIP, may function downstream of PRMT5 to regulate DNA damage signaling and p21-mediated cellular senescence in U2 OS cells. Surprisingly, similar to that of p21 regulation, we also found that knockdown of PRMT5 only upregulated the protein but not the mRNA level of TXNIP.

TRIM21, which belongs to the TRIM family, is an E3 ubiquitin ligase with a RING domain. TRIM21 is widely involved in cell proliferation, differentiation, autophagy, innate immunity and migration [50–53]. Our previous mass spectrometry identified several E3 ligases include CHIP and TRIM21 as interacting proteins of PRMT5 in prostate cancer cells [11]. Consistent with this, a recent study also presented evidence that PRMT5 may functionally interact with TRIM21 in myeloma cells [54]. Consistent with these, we confirmed the interactions between TRIM21 and PRMT5 at both endogenous and exogenous levels in U2 OS cells. Our finding that knockdown of TRIM21 significantly induced senescence in U2 OS cells was particularly interesting, since this finding is the first to delineate the role of TRIM21 in cellular senescence. Our finding is supported by recent findings that higher expression of TRIM21 decreases the response to CDDP in colon cancer and pancreatic cancers, and that somehow contributes to chemo-resistant [55]. We also confirmed that active TRIM21 (but not the RING domain deletion mutant), in cooperation with PRMT5, was involved in the regulation of the TXNIP/p21 axis. These results suggest that overexpressed PRMT5 likely recruits TRIM21 through their physical interaction to facilitate post-translational regulation of TXNIP/p21 expression in OS cells. One possible mechanism is that TRIM21 may ubiquitinate TXNIP and promote TXNIP degradation. Consistent with the role of TXNIP in regulation of DDR [45], knockdown of TXNIP also suppressed the induction of p21 expression by PRMT5 depletion. While this does suggest that the induction of p21 expression may be still dependent on the extent of DNA damage, yet the reason why this regulation is independent of p53 in OS cells remains to be investigated. Alternatively, PRMT5/TRIM21 may also directly regulate the expression of p21 post-translationally. Future studies will investigate whether PRMT5 forms a ternary complex with TRIM21 and TXNIP or p21 and whether PRMT5 may methylate any of these proteins.

In summary, we found that PRMT5 is overexpressed in OS and its overexpression is correlated with OS progression. In addition, we found that PRMT5 plays a key role in regulating senescence of OS cells either in the presence or absence of DNA damaging agents including CDDP. Mechanistically, TRIM21 was found to interact with PRMT5 to regulate senescence in OS cells in response to DNA damage by modulating the expression of TXNIP/p21 (Figure 6H). These findings suggest that overexpression of PRMT5 in OS cells might confer resistance to chemotherapy, e.g. CDDP via modulation of cellular senescence, and that targeting the PRMT5/TRIM21/TXNIP signaling may enhance the therapeutic efficacy in OS.

## MATERIALS AND METHODS

### Cell culture and reagents

Human U2 OS and Saos-2 cells were cultured in McCoy's 5A medium (Life Technologies, Carlsbad, CA, USA) supplemented with 10% or 15% fetal bovine serum, respectively [56]. HEK293T cells were maintained as previously described [10]. CDDP was purchased from Selleck (S1166, TX, USA) and diluted in dimethylformamide (DMF, Dingguo Changsheng Biotechnology Co., Ltd., Beijing, China). Dox was purchased from Selleck (S4163, TX, USA).

### Immunohistochemistry (IHC) staining

Tissue microarray (OS208) containing samples of normal bone and various grades of OS were obtained from Alenabio (Alenabio, Shanxi, China) and used for IHC with an antibody against PRMT5 (79998, 1:400, CST, MA, USA). A total of 34 OS sections were collected from patients at the First Affiliated Hospital of Jinan University and the Third Affiliated Hospital of Sun Yat-sen University. Briefly, paraffin sections were deparaffinized in xylene and rehydrated through graded ethanol washes (100%-70%, v/v), followed by incubation with 3% hydrogen peroxide for 10 min. Antigen retrieval was performed by heating slides in 10 mM Tris-HCl (pH = 10) for 15 min in a microwave. After three washes with phosphate-buffered saline (PBS) containing 0.1% Tween 20 (PBST), slides were blocked in 5% nonfat milk in PBST at room temperature (RT) for 1 h. Slides were incubated with primary antibody against PRMT5 at 4°C overnight, followed by three washes with PBST and incubation with HRP-conjugated anti-rabbit secondary antibodies at RT for 1 h. The signal was developed with diaminobenzidine for 10 min, and sections were counterstained with hematoxylin. Another set of 11 OS fresh sections was used for IHC to correlate the expression of PRMT5 and Ki67 (IS62630-2, Dako, Glostrup, Denmark). Semiquantitative analysis of PRMT5 expression was performed as previously described [17].

### Bioinformatics analysis

The expression of PRMT5 in OS was extracted from the mixed osteosarcoma-Kuijjer dataset in the R2 Genomics Analysis and Visualization Platform (http://r2.amc.nl) [57]. A Kaplan-Meier survival curve was generated to determine the association between PRMT5 expression and patient survival status on the R2 website, at which a large quantity of public genomic data can be accessed for analysis.

### Lentiviral infection of OS cells

Lentiviruses expressing shRNA against PRMT5 (shP5) and TRIM21 (shT21) or SC shRNA were purchased from GenePharma (Shanghai, China). Lentiviruses expressing inducible TRIM21 were generated by co-transfected HEK293T cells with H125 pLenti-TRE-EGFP-EF1-rtTA3-IRES-Puro-TRIM21, pLP1, pLP2, and pLP-VSVG using at a ratio of 1:1:1:2. U2 OS cells were infected with the above lentiviruses by the addition of viral supernatant (multiplicity of infection, MOI = 10) for 24 h and were then selected with 2 μg/ml of puromycin for 3 days. Puromycin (1 μg/ml) was used to maintain stable cells. The targeting sequences of shPRMT5 and shTRIM21 are specified in Supplementary Table 1. The H125-TRIM21-overexpressing stable cells were established and overexpression of TRIM21 was achieved by the addition of Dox as described previously [58].

### SA-β-gal staining

U2 OS cell senescence was detected by Senescence β-Galactosidase Staining Kits (Beyotime, China) according to the manufacturer’s instructions. Briefly, U2 OS cells were seeded into six-well plates and cultured for 72 h. Cells were then fixed for 20 min at RT with 1 ml of fixative solution and were then washed twice with PBS. Next, cells were stained with a staining solution mixture containing X-gal overnight at 37°C.

### Western blot (WB)

Briefly, U2 OS cells were collected and lysed using cell lysis buffer for WB and IP (P0013, Beyotime, Shanghai, China) supplemented with phenylmethylsulfonyl fluoride (PMSF, ST506, Beyotime, Shanghai, China) and protease inhibitor cocktail (Roche, Mannheim, Germany). Proteins were then electrophoresed using SDS-PAGE gels (Beyotime, Shanghai, China) and transferred to polyvinylidene difluoride membranes (PVDF, 0.22 μm, PALL-BSP0161, MD, USA). After blocking, membranes were incubated with primary antibodies and then with HRP-conjugated secondary antibodies at RT. The primary antibodies used were as follows: anti-β-actin (8H10D10, 1:1000; CST, MA, USA), anti-PRMT5 (07-405, 1:1000; Millipore, Darmstadt, Germany), anti-p21 (12D1, 1:1000; CST, MA, USA), anti-PCNA (D3H8P, 1:1000; CST, MA, USA), anti-p16 (92803S, 1:1000; CST, MA, USA), anti-γ-H2A.X (20E3, 1:1000; CST, MA, USA), anti-p53 (9282, 1:1000; CST, MA, USA), anti-TXNIP (D5F3E, 1:1000; CST, MA, USA), anti-TRIM21 (D1O1D, 1:1000; CST, MA, USA), anti-Lamin B1 (D9V6H, 1:1000; CST, MA, USA), and anti-RAD51 (PC130, 1:1000; Merck, Darmstadt, Germany). Secondary HRP-conjugated antibodies (1:1000) were purchased from CST (MA, USA). Immunoreactive bands were visualized using Clarity Western ECL Substrate (Bio-Rad, Hercules, CA, USA).

### Preparation of nuclear and cytoplasmic protein extracts

Cells were plated at a density of 4×10^5^ cells/per dish in 6 cm dishes and incubated for 72 h. For the preparation of nuclear and cytoplasmic protein extracts, NE-PER Nuclear and Cytoplasmic Extraction Reagents (#78833, ThermoFisher Scientific, MA, USA) were used according to the manufacturer’s instructions. Western blot was used to analyze the subcellular localization of p21 and TXNIP.

### Plasmid construction

The coding regions of HA-VC155-TRIM21, TRIM21, and mTRIM21 were amplified from cDNA of OS cells by PCR with Phusion High-Fidelity DNA Polymerase (NEB, MA, USA). The amplification primers used are specified in Supplementary Table 2. The PCR products were then subcloned in-frame into HA-VC155N-Linker-MCS (HA-VC155-TRIM21) or HA-CMV (TRIM21 and mTRIM21) vectors using two enzymatic sites, *EcoRI* and *KpnI*; the plasmids were verified by sequencing and expression analysis.

### DNA damage (comet) assay

A DNA Damage Detection Kit was purchased from KeyGEN BioTECH (KGA240-50), and the experiments were performed according to the manufacturer’s instructions. Cells were harvested and suspended in PBS, mixed with agarose and placed on slides at 4°C for 30 min. Slides were immersed in cold lysis buffer and then incubated with alkaline (300 mM NaOH and 1 mM EDTA, pH = 12.3) for 40 min. After electrophoresis, slides were washed using 0.4 mM Tris-HCl buffer (pH = 7.5) and stained with propidium iodide (PI). DNA damage was then detected using fluorescence microscopy at an excitation wavelength of 515~560 nm. The OTM was analyzed by Open Comet software [59].

### Immunofluorescence

U2 OS cells were cultured on glass coverslips one day before treatment with DMF or CDDP (20 μM) for 3 h, and the medium was then replaced with fresh complete medium for 12 h or 24 h to provide the appropriate conditions for DNA repair. Sides with cells were fixed using 4% paraformaldehyde and permeabilized with 0.2% Triton X-100. After blocking, cells were incubated with primary antibodies (1:200) against γ-H2A.X, 53BP1, RAD51, PRMT5, and TRIM21 for 1 h, followed by incubation with Alexa Fluor 555-labeled anti-rabbit IgG (4413S, Cell Signaling Technology), 4’,6-diamidino-2-phenylindole (DAPI) and phalloidin-Alexa Fluor 573 (Life Technologies) overnight. Images were then acquired using a laser scanning confocal microscope (ZEISS LSM 700, Germany).

### RNA interference

siRNAs targeting different sequences of PRMT5 and TXNIP were used for knockdown experiments (GenePharma, Shanghai, China). Cells were cultured at a density of 2 × 10^5^ cells/well in a 6-well plate (Costar 3516, Corning, NY, USA) overnight. siRNAs targeting PRMT5 and TXNIP were transfected into cells using Lipofectamine 2000 (Invitrogen, CA, USA) for 4 h. Cells were then cultured in fresh complete medium for 3 days. The targeting sequences of the above siRNAs are specified in the supplementary information (Supplementary Table 3).

### BiFC assay

BiFC was performed to analyze the interaction between PRMT5 and TRIM21 in U2 OS cells, as previously described [38, 39]. Briefly, U2 OS cells were cultured on coverslips overnight, followed by cotransfection with plasmids encoding Myc-VN155-PRMT5 and HA-VC155-TRIM21, along with FLAG-Cerulean, for 48 h. Cells were fixed with 4% paraformaldehyde and stained with DAPI at RT for 10 min. Fluorescence images were acquired using a laser scanning confocal microscope (ZEISS LSM 700, Germany).

### Co-immunoprecipitation (Co-IP)

Co-IP assays were performed to explore the endogenous interaction between PRMT5 and TRIM21 in U2 OS cells. Briefly, cells were incubated in lysis buffer (P0013, Beyotime, Shanghai, China) supplemented with NaF, PMSF, Na_3_VO_4_ and protease inhibitors. After incubation on ice for 30 min, 40 μl of 50% protein A agarose bead slurry (CST, MA, USA) was used to preclear cell extracts for 30 min at 4°C, followed by incubation with 5 μg of either IgG (CST, MA, USA) or antibody against PRMT5 overnight at 4°C. Immune complexes were then incubated with 40 μl of 25% protein A-Sepharose slurry for 2 h. Immunoprecipitates were subjected to WB after being washed five times with lysis buffer. Notably, IPKine HRP AffiniPure Mouse Anti-Rabbit IgG Light Chain (A25022, Abbkine, Wuhan, China) was used as the secondary HRP-conjugated antibody to eliminate interference from heavy chain fragments, which have a similar molecular weight as TRIM21.

### Reverse transcription and real-time PCR

Reverse transcription and real-time PCR were performed as described in our previous studies [56]. A TRIzol Plus RNA Purification Kit (Life Technologies) was used to isolate total RNA from U2 OS cells. Purified RNA was then reverse transcribed to cDNA using a High Capacity cDNA Reverse Transcription Kit (Invitrogen, CA, USA) according to the manufacturer’s instructions. For real-time PCR, a CFX96 Touch™ Real-Time PCR Detection System (785BR15759, Bio-Rad, CA, USA) was used with Fast SYBR GREEN Master Mix (Applied Biosystems). Gene expression was reported as the relative fold change (2^−ΔΔCT^) and was normalized to control gene expression. Primers used for real-time PCR are specified in the supplementary information (Supplementary Table 4). The experiments were repeated at least three times, and the results are expressed as the means ± standard deviations (SDs).

### Statistical analysis

All experiments were performed at least three times, and the data are expressed as the means ± SDs. Statistical analysis was performed using GraphPad Prism 6 software (GraphPad Software, San Diego, CA, USA). Comparisons between two groups were performed by using Student's *t*-test. Values of *p*> 0.05 were considered no significant (n.s.); *p*< 0.05 was considered statistically significant (*).

## Supplementary Material

Supplementary Methods

Supplementary Figures

Supplementary Tables
