# Supplementary material for: PRMT5-TRIM21 interaction regulates the senescence of osteosarcoma cells by targeting the TXNIP/p21 axis
Source: Aging (Albany NY). 2020 Feb 5;12(3):2507–29. doi: 10.18632/aging.102760 (PMC7041745; doi:10.18632/aging.102760)
Supplement: Supplementary Methods [file aging-12-102760-s001..pdf]

SUPPLEMENTARY METHODS

Analysis of apoptosis by flow cytometry

U2 OS cells were plated at a density of 4×10^5 cells/per dish in 6 cm dishes before transfected with siCon (siControl) or siPRMT5 for 3 days. Cells were then harvested using trypsinization. For the preparation of flow cytometry, an Annexin V-APC/7-amino-actinomycin D Apoptosis Detection Kit (KeyGEN Biotechnology, Nanjing, China) was used according to the manufacturer's instructions. C6 flow cytometry was used to analyze the apoptotic cells.

EdU (5-Ethynyl-2'-deoxyuridine) incorporation assay

Cells were plated at a density of 5×10^4 cells in 12-well plate and incubated for 72 h. The medium was then replaced with solution of EdU reagent (1:1000) and incubated for another 2 hours. 4 % paraformaldehyde was used to fix the cells, followed by Apollo staining and DNA staining according to the manufacturer's protocol. The images were obtained by fluorescence microscopy.
