# Supplementary material for: PRMT5-TRIM21 interaction regulates the senescence of osteosarcoma cells by targeting the TXNIP/p21 axis
Source: Aging (Albany NY). 2020 Feb 5;12(3):2507–29. doi: 10.18632/aging.102760 (PMC7041745; doi:10.18632/aging.102760)
Supplement: Supplementary Figures [file aging-12-102760-s003..pdf]

SUPPLEMENTARY FIGURES

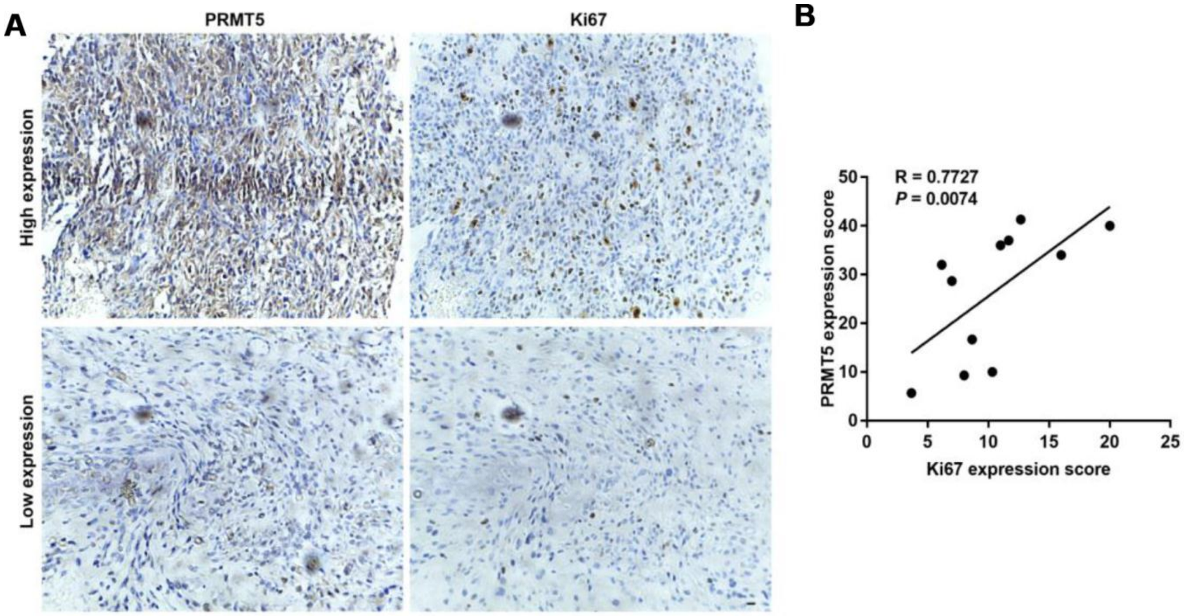

Supplementary Figure 1. PRMT5 expression is positively correlated with the expression of Ki67 in OS tissues. (**A**) 11 OS fresh sections were used for IHC to determine the expression of PRMT5 and Ki67. Scale bar = 30 μm (**B**) The expression score of PRMT5 and Ki67 in A was analyzed. The correlation of PRMT5 and Ki67 was calculated using Spearman rank correlation coefficient.

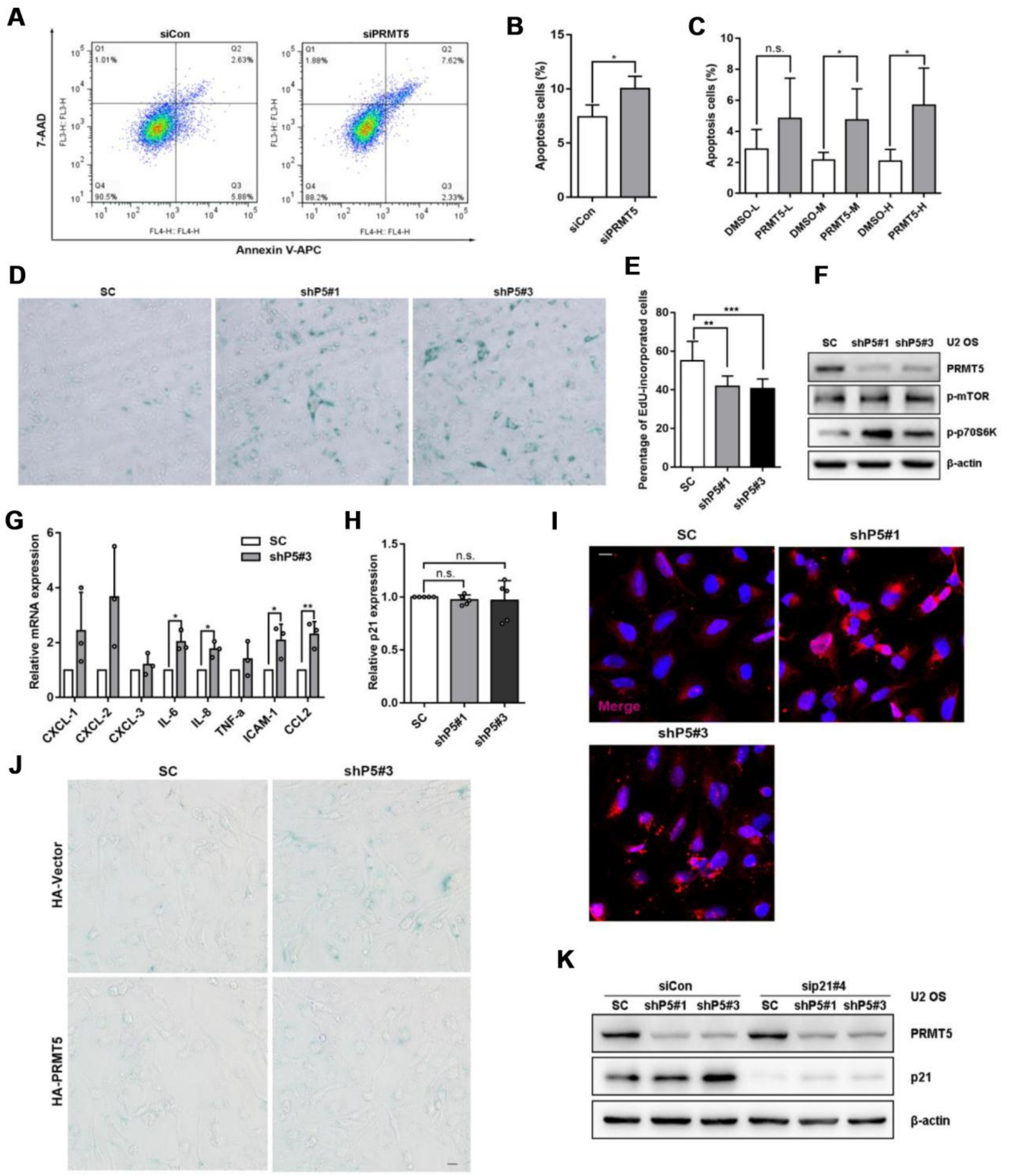

Supplementary Figure 2. Knockdown of PRMT5 causes little cell apoptosis and elevates p21 expression. (**A** and **B**) U2 OS cells were transfected with siCon (siControl) or siPRMT5 for 3 days, then the apoptotic cells were determined by flow cytometry and analyzed using Flowjo software. *, p< 0.05. (**C**) U2 OS cells were treated with different concentrations of EPZ015666 (S7748, Selleck, TX, USA) for 24 h, then the apoptotic cells were determined by flow cytometry and analyzed using Flowjo software. *, p< 0.05. (**D**) Two independent shRNAs targeting PRMT5 (shP5#1 and shP5#3) were applied to knock down PRMT5 expression in Saos-2 cells, and senescent cells were assessed using an SA-β-gal staining kit. Scale bar = 10 μm. (**E**) Two independent shRNAs targeting PRMT5 (shP5#1 and shP5#3) were applied to knock down PRMT5 expression in U2 OS cells, the percentage of EdU-incorporated cells was analyzed. **, p< 0.01; ***, p< 0.001. (**F**) The protein expression of p-mTOR and p-p70 S6K with or without PRMT5 depletion in U2 OS cells was determined by WB. (**G**) The relative expressions of SASP genes were analyzed by quantitative real-time PCR with or without PRMT5 knockdown in U2 OS cells, and the results were obtained from three independent experiments. (**H**) The mRNA expression of p21 with or without PRMT5 depletion was determined by quantitative real-time PCR. (**I**) The antibody against p21 was used for immunofluorescence staining, and the subcellular localization of p21 in U2 OS cells with or without PRMT5 depletion was captured under confocal microscope. Scale bar = 10 μm. (**J**) Plasmids encoding HA-PRMT5 were transfected into the SC or shP5#3 U2 OS cells, and senescent cells were visualized using the SA-β-gal staining kit. Scale bar = 20 μm. (**K**) siRNA targeting p21 (sip21) was transfected into U2 OS cells with or without PRMT5 depletion for 3 days, the protein expression of p21 was determined by WB.

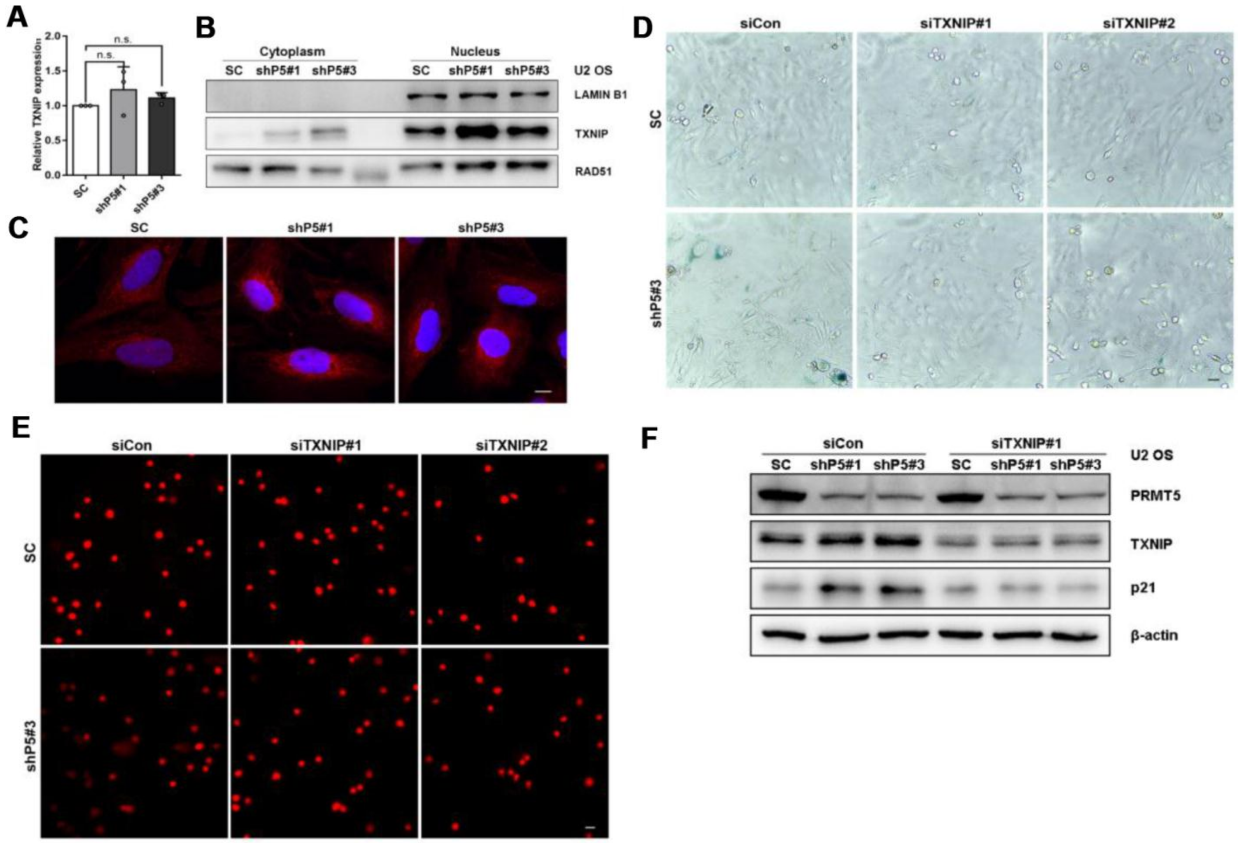

Supplementary Figure 3. The up-regulation of TXNIP by PRMT5 depletion correlated with DSBs. (**A**) The mRNA expression of TXNIP was determined by quantitative real-time PCR with or without PRMT5 knockdown in U2 OS cells. (**B**) Cytoplasmic and nuclear proteins were isolated, and the protein expression level of TXNIP with or without PRMT5 depletion was determined by WB. LAMIN B1 was used as an internal control of nucleus. RAD51 was used as positive control. (**C**) The subcellular localization of TXNIP was observed by immunofluorescence staining under confocal microscope. Scale bar = 10 μm. (**D**) Two independent siRNAs targeting TXNIP (siTXNIP#1 and siTXNIP#2) were transfected into SC or shP5#3 U2 OS cells for 3 days, and senescent cells were visualized using an SA-β-gal staining kit. Scale bar = 10 μm (**E**) siRNAs targeting TXNIP were transfected into the SC and shP5#3 U2 OS cells, the DNA damage was visualized by comet assay. Scale bar = 50 μm. (**F**) siRNA targeting TXNIP was transfected into the SC, shP5#1, and shP5#3 U2 OS cells, the expressions of PRMT5, TXNIP and p21 were determined by WB.
