# Supplementary material for: PRMT5-TRIM21 interaction regulates the senescence of osteosarcoma cells by targeting the TXNIP/p21 axis
Source: Aging (Albany NY). 2020 Feb 5;12(3):2507–29. doi: 10.18632/aging.102760 (PMC7041745; doi:10.18632/aging.102760)
Supplement: Supplementary Tables [file aging-12-102760-s002..pdf]

SUPPLEMENTARY TABLES

Supplementary Table 1. Sequences of shRNAs for PRMT5 and TRIM21.

Target	Sequence
PRMT5#1	GGGACTGGAATACGCTAATTG
PRMT5#3	GGCTGACCTCCCATCTAATCA
TRIM21#1	CAGCACGCTTGACAATGAT
TRIM21#2	GACTTCACCTGTTCTGTGA

Supplementary Table 2. List of the primers used for the plasmids construction.

Target gene	Foward	Reverse
HA-TRIM21	CCGAATTCTCATGGCTTCAGCAGCACGC	CCGGTACCATAGTCAGTGGATCCTTGTGATCC
HA-mTRIM21	GTCGAATTCTCCAGCGCTTTCTGCTCAAGAAT	CCGGTACCATAGTCAGTGGATCCTTGTGATCC
Myc-VC155-TRIM21	CCGAATTCTCATGGCTTCAGCAGCACGC	CCGGTACCATAGTCAGTGGATCCTTGTGATCC
HA-PRMT5	CCGAATTCGGAGAAAGATGGCGGCGATG	CCGCTCGAGCGGGCAGGGCTAGAGGCCAATGGT

Supplementary Table 3. List of the siRNAs sequences targeting PRMT5, p21 and TXNIP.

Target gene	Forward	Reverse
PRMT5#1	CCGGACUUUGUGUGACUAUTT	AUAGUCACACAAAGUCCGGTT
PRMT5#2	CAGCAGGCCAUCUAUAAAUTT	AUUUAUAGAUGGCCUGCUGTT
PRMT5#3	CCAGUUUGAGAUGCCUUAUTT	AUAAGGCAUCUCAAACUGGTT
PRMT5#4	GGUGAACACAGUACUACAUTT	AUGUAGUACUGUGUUCACCTT
p21#4	GGAACAAGGAGUCAGACAUTT	AUGUCUGACUCCUUGUUCCTT
TXNIP#1	GAGACCUGGAAACAAAUAUTT	AUAUUUGUUUCCAGGUCUCTT
TXNIP#2	GUCAGAGGCAAUCAUAUUATT	UAAUAUGAUUGCCUCUGACTT

Supplementary Table 4. List of the primers used for the real-time PCR.

Target gene	Foward	Reverse
CXCL-1	ACTCAAGAATGGGCGGAAAGC	TCAGGAACAGCCACCAGTGAG
CXCL-2	TCGCACAGCCGCTCGAAC	GGGGGACTTCACCTTCACACTT
CXCL-3	GTGTGAATGTAAGGTCCCCCG	ATTTTCAGCTCTGGTAAGGGC
IL-6	AGTGAGGAACAAGCCAGAGC	AGCTGCGCAGAATGAGATG
IL-8	CAGTTTTGCCAAGGAGTGCT	GTTTTCCTTGGGGTCCAGAC
TNF-a	CCAGACCAAGGTCAACCTCC	CAGACTCGGCAAAGTCGAG
ICAM-1	CGACTGGACGAGAGGGATTG	GGAGAGCACATTCACGGTC
CCL2	TCTCAAACTGAAGCTCGCACT	GGGAATGAAGGTGGCTGCTA
p21	AGGGGACAGCAGAGGAAGA	GGCGTTTGGAGTGGTAGAAAT
TXNIP	GCCACACTTACCTTGCCAAT	TGATCTTCTGAACCCGAAGG
TRIM21	CCAATCCGTGGCTGATACTT	GCACCCAGGACCATAGGATA
GAPDH	CCCTGTTGCTGTAGCCAAAT	CTGACTTCAACAGCGACACC
